# Knowledge, Attitudes, and Practices (KAP) Regarding Diabetes-Related Hearing Loss Among Providers and Patients: A Scoping Review

**DOI:** 10.3390/healthcare13233025

**Published:** 2025-11-24

**Authors:** Mehwish Nisar, Muhammad Waqas Nisar Ahmed, Anjana Rajagopal, Beenish Nisar Ahmed, Zohra S. Lassi

**Affiliations:** 1 School of Health and Rehabilitation Sciences, The University of Queensland, Brisbane 4072, Australia; 2 Community Medicine Department, Jinnah Sindh Medical University, Karachi 75510, Pakistan; 3 Department of Otorhinolaryngology and Head & Neck Surgery, Avicenna Medical Complex Hospital, Islamabad 44090, Pakistan; 4 Department of Otorhinolaryngology, College of Medicine, Bahria University, Islamabad 44000, Pakistan; 5 School of Public Health, Faculty of Health and Medical Sciences, The University of Adelaide, Adelaide 5000, Australia; 6 Robinson Research Institute, The University of Adelaide, Adelaide 5000, Australia

**Keywords:** diabetes care, health literacy, long-term complications, healthcare professionals, audiology, hearing screening

## Abstract

**Highlights:**

**What are the main findings?**

- Limited evidence exists examining knowledge, attitudes, and practices regarding diabetes-related hearing loss, with only a few studies identified from four countries, focusing predominantly on healthcare providers with minimal patient data.
- Knowledge of diabetes-related hearing loss was substantially lower among both providers and patients compared to other complications, reflected in poor screening attitudes, minimal practices and counseling, and hearing screening rates.

**What are the implications of the main finding?**

- Systematic integration of hearing evaluations into diabetes care pathways offers potential for improved patient outcomes and reduced undiagnosed complications, though implementation strategies require further investigation.
- Future research should prioritise standardised KAP assessment instruments, large-scale educational intervention studies across diverse settings, qualitative investigations of patient and provider perspectives, and cost-effectiveness analyses to inform policy development.

**Abstract:**

Background: Hearing loss remains significantly underrecognised as a diabetes complication, despite diabetic individuals experiencing double the risk of sensorineural hearing impairment. This review synthesised healthcare provider and patient knowledge, attitudes, and practices (KAP) concerning diabetes-related hearing loss. Methods: Using Arksey and O’Malley’s framework and PRISMA-ScR guidelines, systematic searches were conducted across five databases (PubMed/MEDLINE, Embase, Scopus, Web of Science, Cochrane Library) through August 2025. Grey literature and reference screening supplemented database searches. Eligible studies examined KAP among healthcare professionals and diabetic patients. Results: Five studies from four countries reported KAP findings were identified from 2029 records, encompassing 2813 healthcare providers. Only one study included KAP data from patients. Knowledge deficits were pronounced: American provider awareness ranged 25.6–44.5%, contrasting sharply with >94% awareness of other complications. Only 24.3% of Chinese providers demonstrated complete understanding, while 59.7% of South African practitioners remained unaware of auditory complications. Patient knowledge was similarly limited (21% recognition). Attitudes showed moderate engagement, with 68–75% of Chinese practitioners accepting management responsibilities. Barriers included unfamiliar guidelines, competing priorities, and restricted audiological access. Practices were suboptimal: 16.9% conducted routine screening, referrals remained reactive, and 64.9% never discussed hearing risks. Training opportunities were minimal. Conclusions: Substantial KAP deficits exist regarding diabetes-related hearing loss. Recognition disparities compared to established complications represent lost opportunities for early intervention. Urgent needs include standardised assessment instruments, large-scale intervention studies, and comprehensive educational programs to integrate hearing health into standard diabetes management protocols.

## 1. Introduction

Diabetes mellitus (DM) and hearing loss represent two pervasive global health challenges, collectively impacting billions and significantly augmenting public health burdens. The International Diabetes Federation projects DM to affect 589 million adults globally, a figure anticipated to rise to 853 million by 2050 [1], while the World Health Organisation (WHO) estimates that over 1.5 billion people live with some degree of hearing loss [2]. The convergence of these conditions is not merely coincidental but mechanistically linked; individuals with DM face approximately double the risk of developing sensorineural hearing loss. A recent meta-analysis demonstrated that significant hearing impairment affected 53% of individuals with Type 2 diabetes, in contrast to 25% of non-diabetic individuals (RR = 2.3). Within the diabetic population, 15% exhibited unilateral loss and 29.6% presented with bilateral loss [3].

Despite the robust and accumulating evidence, hearing loss remains significantly under-recognised within routine diabetes care pathways. Provider and patient awareness play a crucial role in early detection and management [4]. When healthcare professionals lack knowledge of the auditory consequences of diabetes, screening is rarely initiated, counselling is limited, and referrals are inconsistent. Equally, patients with low awareness may not recognise symptoms, seek timely care, or understand the importance of hearing surveillance. These awareness gaps collectively contribute to delayed diagnosis, untreated impairment, and preventable communication and cognitive difficulties. Despite strong epidemiological links, the integration of knowledge, attitudes, and practices (KAP) concerning diabetes-related hearing loss into routine clinical care and patient education remains notably suboptimal [4,5].

The pathophysiological underpinnings of this association are complex and multifactorial, drawing on established theories of microvascular disease and neurodegeneration [6]. Chronic hyperglycaemia is hypothesised to instigate cochlear microangiopathy, leading to structural and functional damage within the delicate microvasculature of the inner ear. It also fuels oxidative stress, harming cochlear hair cells and neurons [4]. Furthermore, diabetes can cause auditory neuropathy, impairing neural firing in the auditory pathway via demyelination or degeneration [7]. These mechanisms accelerate cochlear aging, resulting in earlier and more severe hearing decline [8]. Adding to this, certain medications prescribed for diabetes management or its comorbidities, notably ototoxic loop diuretics like furosemide (used for conditions like hypertension or diabetic nephropathy), can exacerbate auditory damage [9].

Despite substantial evidence linking diabetes to an increased risk of hearing loss, this complication remains largely absent from standard clinical guidelines [10]. This gap is particularly concerning given the global prevalence of diabetes and the profound impact hearing loss has on daily functioning and overall well-being. Yet, the limited guideline recognition represents only part of the challenge. The more pressing issue lies in the real-world consequences for affected individuals. Diabetes-related hearing loss can hinder communication with healthcare providers, reduce a patient’s ability to understand verbal health instructions, compromise diabetes self-management, and heighten risks of social isolation, depression, and cognitive decline [8]. Together, these impacts highlight the critical need to recognise, assess, and manage hearing loss as an integral component of comprehensive diabetes care.

Healthcare providers, often time-constrained, prioritise well-established complications like retinopathy, nephropathy, and foot ulcers. This creates a disparity: traditional complications are universally screened and discussed, while auditory issues are rarely acknowledged as diabetes related [6]. Patients similarly lack awareness, receiving extensive counselling on visual and renal risks but little on hearing consequences. This oversight is critical given hearing loss’s profound impact on communication, cognition, social engagement, and quality of life, potentially increasing diabetes’s psychosocial burden [8]. This gap leads to reduced participation in hearing health services, delayed care-seeking, and missed opportunities for preventive intervention—further reinforcing the need to understand current levels of awareness and practice among stakeholders.

This profound disconnect between established scientific evidence and routine clinical practice perpetuates a cycle of under-screening, delayed diagnosis, and inadequate management of a potentially preventable or mitigable complication [10]. Consequently, substantial patient populations remain vulnerable to a comorbidity that significantly compromises their well-being [11]. While extensive research has elucidated the prevalence rates and underlying mechanisms of diabetes-related hearing loss, a comprehensive synthesis systematically examining the knowledge, attitudes, and practices (KAP) of healthcare providers and patients is conspicuously lacking [12]. To our knowledge, this review represents the first systematic synthesis of KAP related to diabetes-associated hearing loss, addressing a critical evidence gap and establishing a foundation for informed clinical, educational, and policy interventions.

This scoping review systematically examines existing evidence on knowledge, attitudes, and practices regarding diabetes-related hearing loss among healthcare providers and patients. Specifically, we investigate: the type of available studies; the extent of current knowledge and awareness; reported attitudes and practices; and assessment instruments employed. This mapping of the evidence landscape will identify research gaps and inform policy development for integrating hearing health into diabetes care.

## 2. Materials and Methods

A scoping review methodology was selected as most appropriate given our exploratory aims to map the breadth and nature of existing evidence across diverse study designs, populations, and assessment instruments. Unlike systematic reviews that evaluate intervention effectiveness, scoping reviews are specifically designed to examine emerging topic areas, accommodate methodological heterogeneity, and identify research gaps to guide future inquiry [13,14].

### 2.1. Identifying the Research Question

This scoping review was guided by four primary research questions: (1) What is the extent and nature of studies examining KAP related to diabetes-related hearing loss among doctors, audiologists, and patients? (2) What is the extent and depth of healthcare provider and patient knowledge regarding diabetes-associated hearing loss? (3) What attitudes and practices regarding diabetes-related hearing loss screening and management are reported by healthcare providers and patients, and what barriers or facilitators influence these practices? (4) What instruments and methodological approaches have been employed to assess KAP dimensions related to diabetes-associated hearing loss?

### 2.2. Identifying Relevant Studies

This scoping review adhered to the methodological framework established by Arksey and O’Malley [13] and followed the Preferred Reporting Items for Systematic Reviews and Meta-Analyses extension for Scoping Reviews (PRISMA-ScR) guidelines (See Table S1) [14]. The review is registered in Open Science Framework (https://osf.io/s9teh, accessed on 16 November 2025). A comprehensive search strategy was developed in consultation with an expert librarian and executed across five electronic databases: PubMed/MEDLINE, Embase, Scopus, Web of Science, and the Cochrane Library. These databases were selected for their relevance to health sciences and multidisciplinary coverage. A comprehensive search strategy was developed to identify studies examining knowledge, attitudes, and practices (KAP) regarding diabetes-related hearing loss. The strategy combined controlled vocabulary and free-text terms relating to three core domains: (1) diabetes (e.g., “diabetes mellitus”, “diabetic”), (2) hearing loss (e.g., “hearing impairment”, “auditory”), and (3) KAP constructs (e.g., “awareness”, “knowledge”, “attitudes”, “practices”, “survey”). Boolean operators were applied to ensure comprehensive coverage. Database-specific adaptations were undertaken to reflect individual indexing systems and syntax requirements. For instance, Medical Subject Headings (MeSH) were incorporated in PubMed (e.g., “Health Knowledge, Attitudes, Practice”), while equivalent thesaurus terms were employed in Embase, Scopus, Web of Science, and the Cochrane Library. Free-text keywords were used in conjunction with controlled terms to optimise sensitivity (See Table S2). The search was conducted on 1 August 2025, without date or language restrictions to ensure comprehensive identification of all relevant evidence. Given the emerging nature of KAP research specifically examining diabetes-related hearing loss, we prioritised exhaustive retrieval to capture the full extent of available evidence. Non-English records were assessed using web-based translation tools (Google Translate, DeepL) to minimise exclusion of relevant international studies. Grey literature searches were performed using Google Scholar, ProQuest Dissertations and Theses, and relevant organisational websites, including diabetes associations, audiology societies, and public health agencies. Additionally, reference lists of included studies underwent manual screening to identify potentially relevant sources. Some studies, particularly grey literature sources and professional association surveys, are not indexed in Web of Science, which explains why they may not appear in WOS searches. We therefore clarify that although WOS was searched, most relevant records were retrieved through PubMed, Embase, Scopus, and grey literature sources.

### 2.3. Study Selection

Study selection criteria were established using the Population-Concept-Context (PCC) framework [15]:

- Population: Healthcare providers, including general practitioners, endocrinologists, diabetologists, diabetes educators, and other diabetes care specialists; audiologists; and patients diagnosed with type 1 or type 2 diabetes mellitus.
- Concept: Knowledge, awareness, attitudes, or practices concerning diabetes-associated hearing loss and its management.
- Context: Primary research studies employing quantitative, qualitative, or mixed methods designs; reports; theses; and policy documents published in English from 2000 onwards.

Exclusion criteria: Studies focusing exclusively on biomedical associations between diabetes and hearing loss without KAP components; case reports; editorials; commentary pieces; and non-English language publications.

### 2.4. Study Selection Process

All retrieved records were imported into Covidence, a web-based systematic review management platform. Two independent reviewers (MN and MW) conducted screening in two sequential phases: title and abstract screening followed by full-text evaluation. Disagreements at any stage were resolved through discussion, with a third reviewer consulted when consensus could not be reached. The study selection process is illustrated in a PRISMA flow diagram.

### 2.5. Charting the Data

A standardised data extraction form was developed, piloted on a subset of studies, and refined before implementation. Two reviewers (MN and AR) independently extracted data elements for each included study, encompassing bibliographic details (authors, publication year, country, journal), study design and methodological approach, setting and context, population characteristics and sample size, KAP assessment instruments and methodologies, and primary findings organised by KAP domains. Data extraction discrepancies were resolved through discussion and consensus, with extracted data organised systematically and reviewed by the research team to ensure accuracy and completeness. In accordance with established scoping review methodology, formal quality assessment of included studies was not conducted.

### 2.6. Synthesis of Results

**Figure 1:**
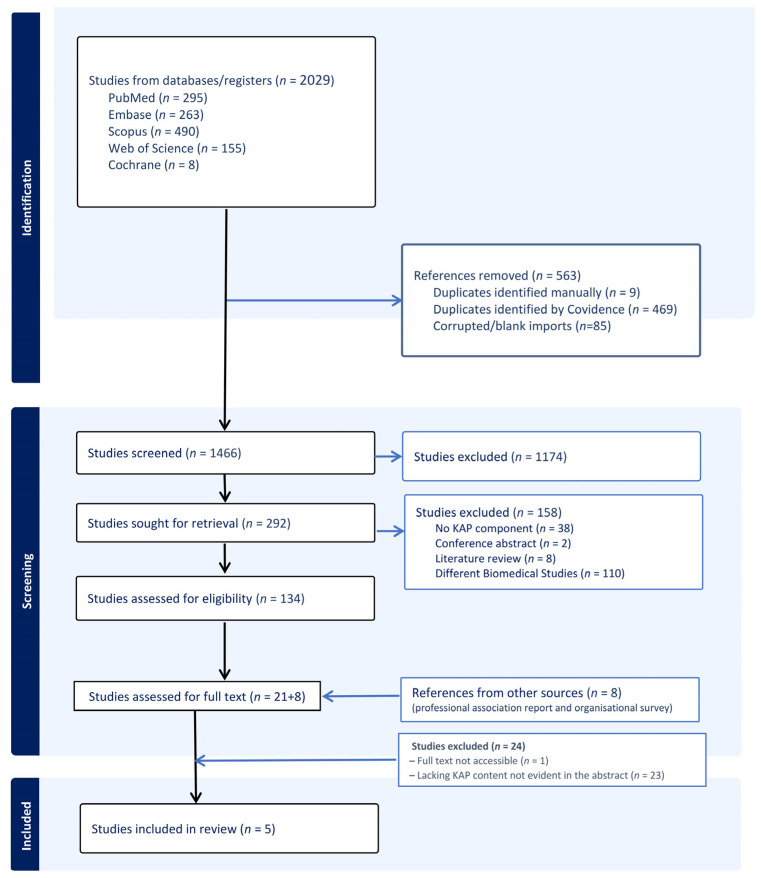
PRISMA Flow Diagram of Study Selection Process.

Results were synthesised using descriptive analytical approaches encompassing four key components. First, a comprehensive summary provided an overview of study designs, geographical distribution, settings, and participant characteristics. Second, thematic organisation involved systematic categorisation of findings according to KAP domains across stakeholder groups. Third, methodological analysis comprised a comprehensive review of instruments and approaches used for KAP assessment. Fourth, an identification of gaps in existing research was conducted to highlight areas requiring further investigation. Findings are presented through narrative synthesis supported by a comprehensive summary detailing study characteristics and key results across KAP domains. To support analytical transparency, we adopted a structured narrative synthesis informed by Arksey & O’Malley and JBI guidance. Three reviewers (MN, MW, AR) collaboratively charted data, organised findings into predefined KAP domains, and refined themes through iterative meetings. Figure 1 was developed through team consensus.

## 3. Results

### 3.1. Screening Results

The comprehensive search strategy yielded 2029 records, with duplicates removed before title and abstract screening (Figure 1). Following full-text review, only five studies met the eligibility criteria, comprising four peer-reviewed articles and one grey literature source [16,17,18,19,20]. This limited yield reflects the paucity of research specifically addressing KAP regarding diabetes-related hearing loss. The comprehensive search across multiple databases and grey literature sources suggests this represents a genuine literature gap rather than methodological limitations, underscoring the need for further KAP research among healthcare providers and patients. The majority of excluded records focused exclusively on biomedical associations between diabetes and hearing loss without addressing stakeholder KAP.

### 3.2. Characteristics of the Included Studies

The included studies were conducted across three global regions: the United States (US), South Africa, and China. All adopted a cross-sectional survey design (See Table S3). Stakeholder groups encompassed general medical practitioners, diabetes educators, certified specialists, and mixed providers, as well as patients with diabetes. Although some reports described age, work experience, or professional backgrounds, ethnicity and demographic details were inconsistently reported, limiting insights into how awareness and practices may vary across diverse populations. The studies were published between 2011 and 2025, with the majority (*n* = 4) published within the last five years, indicating growing research interest in this area. Sample sizes ranged from 236 to 1022 participants, with a total of 2813 healthcare providers across all primary research studies.

### 3.3. Instruments and Methods Used to Assess KAP

The included studies employed a range of methodologies to assess KAP, with notable variability in instrument development and validation. Most relied on investigator-developed questionnaires, though only a few described rigorous validation processes. A comprehensive 39-item tool was developed through literature review, stakeholder interviews, and Delphi consultations, achieving strong test–retest reliability (r = 0.863), with the survey assessing KAP across structured domains using validated scoring scales [18]. An 18-item survey was validated through expert content review (I-CVI > 0.79) and pilot testing with practitioners [19], while a 39-item online survey combined multiple-choice, true/false, and open-ended questions [20]. Other studies reported limited instrument details.

### 3.4. Knowledge and Awareness of Diabetes-Related Hearing Loss

**Figure 2:**
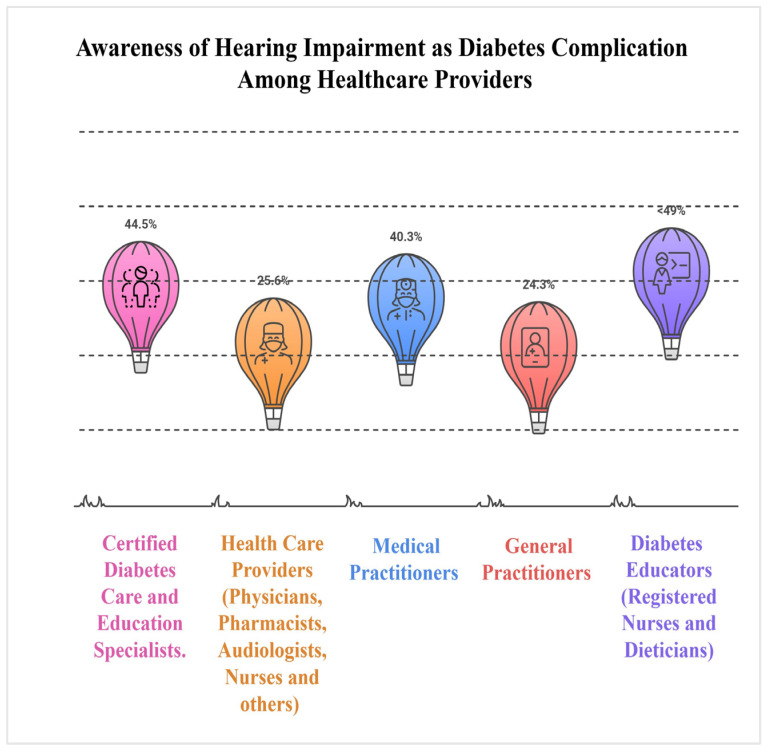
Awareness of diabetes-related hearing loss among healthcare provider groups [16,17,18,19,20].

Across studies, knowledge and awareness of diabetes-related hearing loss were consistently low or incomplete (Figure 2). In the US, only 25.6% of healthcare providers identified hearing impairment as a diabetes complication [17]. Patient awareness was equally limited, with just 21% recognising the ear as affected by diabetes and only 8.1% recalling being informed of this risk by a healthcare provider [17]. Similar gaps were observed, with only 44.5% of certified U.S. diabetes educators acknowledging the association between diabetes and hearing loss [16]. More than half of diabetes educators demonstrated little or no awareness of the association [20]. In South Africa, 59.7% of medical practitioners were unaware of auditory symptoms, with the remainder most frequently identifying hearing loss, tinnitus, nerve damage, infections, and vertigo [19]. A mean knowledge score of 69.9/100 was reported among Chinese general practitioners, although only 24.3% achieved a full score and more than 30% were unfamiliar with screening tools [18].

### 3.5. Attitudes and Perceptions Regarding Hearing Loss in Diabetes Care

Attitudinal findings across studies revealed ambivalence, misconceptions, and competing clinical priorities. U.S. providers cited unfamiliarity with screening guidelines (57.3%) and competing demands (35.4%) as key barriers to referral [17]. Similar challenges were identified, with more than 60% of certified diabetes educators lacking knowledge of referral processes [16]. These gaps were reinforced, though potential was highlighted with nearly 40% of educators expressing willingness to adopt screening tools if training were available [20]. In South Africa, 44.9% of practitioners were unfamiliar with the audiologist’s role in diabetes care, underscoring a critical gap in interdisciplinary awareness [19].

The most detailed attitudinal data revealed a mean score of 66.1/100 among Chinese general practitioners [18]. While 67.2% considered age-related hearing loss a disease and 68–75% agreed it was their duty to manage it, misconceptions persisted, with 11.6% perceiving it as a normal aging process [18]. Only 35.8% believed screening offered economic benefits, and attitudes toward treatment effectiveness were largely neutral [18]. Collectively, these studies indicate that while awareness of responsibility is emerging, hearing health remains undervalued within diabetes care pathways.

### 3.6. Practices: Screening and Referral

**Figure 3:**
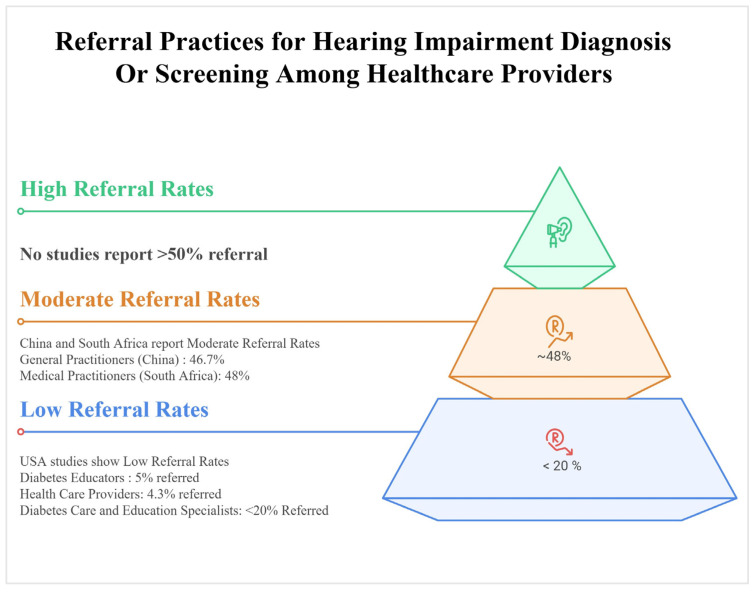
Reported Healthcare Provider Referral Rates for Hearing Impairment Screening and Diagnosis Gaps in the Existing Literature [16,17,18,19,20].

Reported practices consistently showed very low rates of screening and referral for hearing loss compared with other diabetes complications (Figure 3). In the US, only 24.2% of patients had ever undergone hearing screening, compared with 96% receiving regular dilated eye exams [17]. Similar trends were observed in China, where just 16.9% of general practitioners routinely screened for age-related hearing loss, while 10.8% reported never screening at all [18]. Referral rates were equally low: 95% of U.S. diabetes educators reported rarely or never referring patients for audiological evaluation, while over half of U.S. educators in 2025 had referred <20% of their patients [16]. In South Africa, 51.5% of practitioners never referred patients for auditory symptoms, and nearly two-thirds never counselled patients on hearing loss [19]. Practices often relied on symptom-based triggers, such as tinnitus or difficulty hearing in noisy environments, rather than routine screening. Importantly, higher knowledge was positively associated with both attitudes (OR = 2.305, *p* < 0.001) and practices (OR = 1.409, *p* < 0.001), suggesting that improving awareness could directly enhance practice [18]. These findings point to a clear gap: despite robust evidence linking diabetes and hearing loss, systematic integration of hearing screening into diabetes care remains absent across healthcare systems.

**Figure 4:**
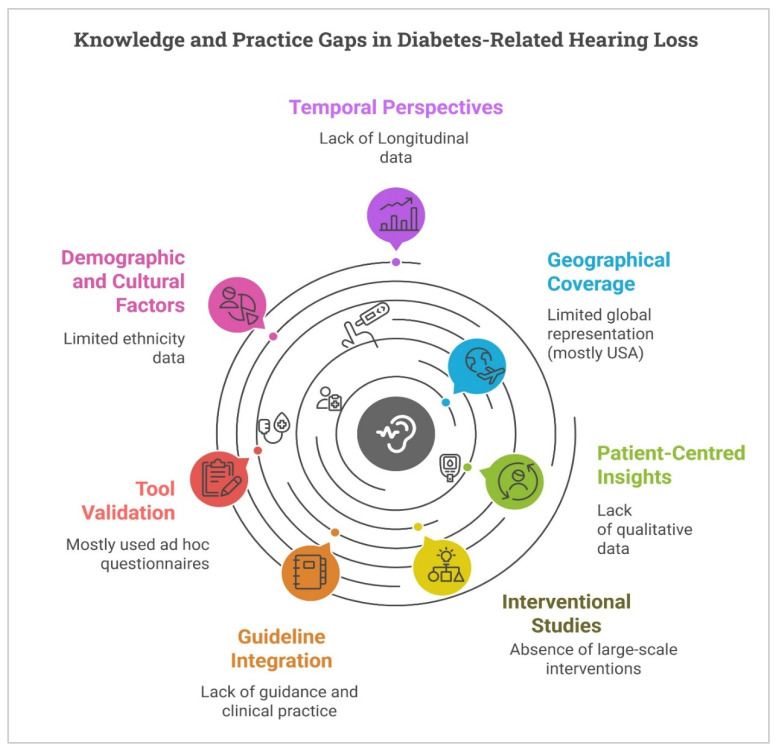
Gaps in the Literature on Knowledge, Attitudes, and Practices (KAP) regarding Diabetes-Related Hearing Loss.

The existing literature on KAP concerning diabetes-related hearing loss reveals significant gaps. Most evidence originates from the United States, limiting global applicability. Patient perspectives are severely underrepresented, with only one study incorporating patient voices and no qualitative research on lived experiences or caregiver roles, thus obscuring real-world barriers to care. Interventional research is notably scarce, comprising one small educational study and no large-scale trials to improve KAP or integrate hearing assessments into diabetes care. Awareness and uptake of recent, inconsistently applied policies are rarely assessed, perpetuating a theory-practice divide. Methodological weaknesses include the prevalent use of unvalidated questionnaires, hindering data comparability. Demographic and cultural factors, such as ethnicity and socioeconomic status, are poorly characterised, preventing analysis of their influence on KAP and health disparities. The exclusively cross-sectional design precludes understanding of longitudinal changes in KAP. Finally, limited examination of diverse practice settings (e.g., rural or resource-limited) restricts a comprehensive understanding of varied healthcare contexts (Figure 4).

## 4. Discussion

This scoping review critically illuminates a significant and persistent knowledge-practice gap concerning diabetes-related hearing loss among both healthcare providers and patients globally. While substantial epidemiological and mechanistic research establishes clear links between diabetes mellitus and auditory complications, hearing loss remains significantly under-recognised compared to established complications such as retinopathy, nephropathy, and neuropathy. Our findings reveal systematic deficiencies, including inadequate stakeholder awareness, suboptimal screening practices, and failure to integrate hearing health into comprehensive diabetes management.

Healthcare providers across diverse settings demonstrate concerning knowledge gaps regarding diabetes-related hearing loss. General practitioners, audiologists, and diabetes specialists frequently exhibit a limited understanding of auditory complications, resulting in the rare incorporation of hearing assessments into routine clinical encounters. This mirrors patient reports of minimal counselling about hearing risks associated with their condition. Despite emerging clinical guidelines advocating hearing surveillance, such as the CDC recommendations for baseline and annual hearing assessments in diabetes patients, practical implementation remains sporadic and inconsistent across healthcare systems, highlighting critical translational deficits [21].

The evolving and contradictory nature of clinical guidelines compounds this uncertainty. The American Diabetes Association has demonstrated an inconsistent stance, omitting hearing loss references until 2021, briefly removing them in 2023, and then reinstating cautious screening recommendations in 2024 [22]. This vacillation reflects the absence of a strong, widely accepted consensus on screening urgency and methodology. Conversely, organisations like the WHO and Endocrine Society have consistently advocated routine hearing monitoring as integral to comprehensive diabetes care [23]. This heterogeneity directly contributes to clinical practice variability and provider confusion, impeding standardised care delivery.

The identified challenges stem primarily from structural and informational deficits within healthcare systems rather than provider negligence. Current practice follows predominantly reactive models, with audiology referrals typically initiated only after patients present with significant, noticeable hearing loss [24]. Evidence from diverse global contexts, including studies from South Africa, North America, and Asia, consistently confirms that preventive hearing screening is rarely embedded in primary diabetes care protocols [23]. This systematic gap forces patients to self-refer after incidental audiology exposure or symptom development, underscoring urgent needs for robust, provider-initiated screening programs [25].

Pragmatic barriers, including resource constraints, limited financial allocations, insufficiently trained personnel, and competing clinical priorities within compressed consultation timeframes, impede widespread screening implementation [26]. Healthcare providers face multiple recommended screening tests for diabetes complications, necessitating evidence-based prioritisation strategies [25]. The current paucity of robust cost-effectiveness data specifically for diabetes-related hearing loss screening compounds these challenges, making it difficult for providers and policymakers to justify additional assessments without clear economic justification or demonstrable patient benefit [4].

Despite significant barriers, promising integration pathways are developing. Innovative programs like The Audiology Project’s ADA collaboration [27] demonstrate successful educational initiatives using accessible tools such as CDC-supported smartphone-based hearing screening [21]. Targeted workshops have notably improved practitioners’ willingness to screen and refer; however, these isolated efforts highlight only one component of a broader evidence gap and should not be viewed as stand-alone solutions. Our findings highlight that while standardised KAP instruments and educational approaches are useful, they are insufficient to fully capture or address the complexities of diabetes-related hearing loss. Qualitative and mixed-methods research is essential to understand real-world patient experiences, communication challenges, cultural influences, and feasibility barriers faced by providers. Such methods can support the development of practical, context-specific, and patient-centered solutions beyond what survey-based tools can offer. Multidisciplinary care models also present important opportunities, with experts advocating systematic protocols linking diabetes and audiology services. In parallel, technology-enabled tools—particularly validated mobile hearing assessment applications—offer scalable and cost-effective ways to expand screening access, especially in underserved settings [21].

Our review identifies substantial knowledge–practice deficits in diabetes-related hearing loss, revealing broader methodological and conceptual gaps. Critically, similar to other emerging comorbidity research, the evidence remains geographically concentrated in high-income countries [28]. This significantly restricts generalisability to low- and middle-income regions where diabetes and hearing loss are rising most rapidly [29]. Furthermore, unlike more established areas where intervention trials span diverse populations, diabetes-related hearing loss research conspicuously lacks evaluations of strategies to improve awareness, screening, or management across patient and provider groups. This underdevelopment mirrors early stages of other under-recognised complications, where guideline adoption lagged until stronger foundational evidence emerged [10]. Collectively, these gaps underscore the immaturity of the diabetes-related hearing loss evidence base and the need for foundational qualitative inquiry, co-design methodologies, and implementation research before targeted interventions can be appropriately developed or recommended.

## 5. Strengths and Limitations of This Review

This review provides the first systematic synthesis of KAP related to diabetes-related hearing loss, addressing a critical gap in the literature. Adherence to PRISMA-ScR guidelines enhances the rigour and reliability of findings. However, some limitations must be acknowledged. The small number of included studies restricts the robustness of conclusions and precludes meta-analysis. The search strategy, focused primarily on peer-reviewed databases, may be subject to publication bias, as studies with negative or null findings regarding KAP interventions may be underrepresented in published literature. Most available evidence originates from the United States, limiting generalisability to diverse health systems and cultural contexts. The exclusion of non-English language studies may have resulted in omission of relevant international evidence. Additionally, the heterogeneity in study designs and predominant use of investigator-developed questionnaires with variable validation limit comparability of findings across studies.

### Implications and Future Directions

Future research should move beyond descriptive KAP surveys toward qualitative, mixed-methods, and co-design approaches that uncover practical needs, barriers, and contextual determinants of care. While educational interventions may ultimately contribute to improved practice, the current evidence does not support specific models, and such interventions should be conceptualised as long-term goals following initial qualitative and implementation-focused work. Multi-country studies are essential to gain a comprehensive understanding of global patterns and regional disparities in diabetes-related hearing loss recognition and management, while longitudinal studies in single countries would be equally crucial for examining how awareness and practices evolve over time. The development of standardised KAP instruments remains important but should be complemented—not replaced—by qualitative methodologies that directly engage patients and providers in defining priorities and feasible pathways forward. Cost-effectiveness analyses of screening and early intervention strategies are also vital to address provider concerns regarding resource allocation, competing clinical priorities, and to provide evidence-based justification for policy changes.

## 6. Conclusions

This scoping review identifies significant opportunities to improve diabetes-related hearing loss recognition through targeted education, systematic integration into routine care, and evidence-based policy development. The limited included studies reflect genuine research scarcity rather than search limitations, highlighting critical investigation needs. While current practice falls short of guidelines and scientific evidence, successful educational interventions demonstrate potential for systematic improvements. Addressing research gaps through rigorous studies will be essential for advancing comprehensive diabetes care and bridging the persistent evidence-practice divide in diabetes-related hearing loss management.

## Data Availability

Not applicable.

## Supplementary Materials

The following supporting information can be downloaded at https://www.mdpi.com/article/10.3390/healthcare13233025/s1. Table S1: Preferred Reporting Items for Systematic reviews and Meta-Analyses extension for Scoping Reviews (PRISMA-ScR) Checklist. Table S2: Search strategy for studies investigating hearing screening and diabetes across five electronic databases (search conducted 1 August 2025). Table S3. Characteristics of included studies on KAP regarding diabetes-related hearing loss. Reference [30] is cited in the supplementary materials.
