# Supplementary material for: Knowledge, Attitudes, and Practices (KAP) Regarding Diabetes-Related Hearing Loss Among Providers and Patients: A Scoping Review
Source: Healthcare (Basel). 2025 Nov 24;13(23):3025. doi: 10.3390/healthcare13233025 (PMC12692358; doi:10.3390/healthcare13233025)
Supplement: Supplementary file 1 [file healthcare-13-03025-s001.zip › healthcare-3926682-supplementary.pdf]

Supplementary

Table S2: Search strategy for studies investigating hearing screening and diabetes across five electronic databases (search conducted 1ST August 2025)

Database	Date Searched	Search Strategy (as executed)	Results
PubMed/MEDLINE	1 August 2025	("Diabetes Mellitus"[Mesh] OR diabetes mellitus[tiab] OR diabetic*[tiab] OR "Prediabetic State"[Mesh] OR prediabetes[tiab] OR "impaired glucose tolerance"[tiab]) AND ("Hearing Loss"[Mesh] OR hearing loss[tiab] OR hearing impairment*[tiab] OR deafness[tiab] OR auditory disorder*[tiab] OR sensorineural hearing loss[tiab] OR ("hearing screening" OR "screening, auditory" OR "auditory screening" OR "hearing screen*" OR "auditory screen*" OR "audiological screen*" OR "audiometric screening" OR "audiometric assessment" OR (hear* AND "health check-up"))) AND ("Health Knowledge, Attitudes, Practice"[Mesh] OR (knowledge OR attitude* OR practice* OR awareness OR KAP OR survey*))	295
Embase	1 August 2025	('diabetes mellitus'/exp OR 'diabetes mellitus':ti,ab OR diabetic*:ti,ab OR 'prediabetic state'/exp OR prediabetes:ti,ab OR 'impaired glucose tolerance':ti,ab) AND ('hearing loss'/exp OR 'hearing disorder'/exp OR 'deafness'/exp OR hearing loss:ti,ab OR hearing impairment*:ti,ab OR auditory disorder*:ti,ab OR sensorineural hearing loss:ti,ab OR ("hearing screening" OR "screening, auditory" OR "auditory screening" OR "hearing screen*" OR "auditory screen*" OR "audiological screen*" OR "audiometric screening" OR "audiometric assessment" OR (hear* AND "health check-up"))) AND ('knowledge'/exp OR 'attitude'/exp OR 'health practice'/exp OR (knowledge OR attitude* OR practice* OR awareness OR KAP OR survey*))	263
Scopus	1 August 2025	TITLE-ABS-KEY((diabetes OR "diabetes mellitus" OR diabetic* OR prediabetes OR "chemical diabetes" OR "latent diabetes" OR "prediabetic state" OR "impaired glucose tolerance")) AND TITLE-ABS-KEY(("hearing loss" OR "hearing impairment" OR deafness OR "auditory disorder" OR "sensorineural hearing loss" OR {hearing_block})) AND TITLE-ABS-KEY({KAP_block})	490
Web of Science	1 August 2025	TS=((diabetes OR "diabetes mellitus" OR diabetic* OR prediabetes OR "chemical diabetes" OR "latent diabetes" OR "prediabetic state" OR "impaired glucose tolerance")) AND TS=("hearing loss" OR	155

		"hearing impairment" OR deafness OR "auditory disorder*" OR "sensorineural hearing loss" OR {hearing_block}) AND TS=((knowledge OR attitude* OR practice* OR awareness OR KAP OR survey*))	
Cochrane Library	1 August 2025	((diabetes OR "diabetes mellitus" OR diabetic* OR prediabetes OR "chemical diabetes" OR "latent diabetes" OR "prediabetic state" OR "impaired glucose tolerance")) AND ("hearing loss" OR "hearing impairment" OR deafness OR "auditory disorder" OR "sensorineural hearing loss" OR ("hearing screening" OR "screening, auditory" OR "auditory screening" OR "hearing screen*" OR "auditory screen*" OR "audiological screen*" OR "audiometric screening" OR "audiometric assessment" OR (hear* AND "health check-up"))) AND ((knowledge OR attitude* OR practice* OR awareness OR KAP OR survey*))	8

Notes:

- TS searches Title, Abstract, Author Keywords, and Keywords Plus.
- Because WOS indexing is narrower for KAP-style terminology, many relevant articles appear only when "survey", "knowledge", or "awareness" is included as a free-text word.
- The relevant diabetes–hearing KAP records identified in our review came from grey literature and did not always appear in WOS, which is why the reviewer did not retrieve them.
- No date or language restrictions applied.

Table S3. Characteristics of included studies on KAP regarding diabetes-related hearing loss.

Study	Country / Setting	Population (Stakeholder Group) / Demographic Characteristics	Design / Instruments Used	KAP Components Assessed by Included Studies	Key KAP Findings
Zelnicek et al. (2025)	USA (nationwide survey)	Certified Diabetes Care and Education Specialists (nurses, pharmacists, dietitians). National sample: ethnicity not reported.	Cross-sectional survey / Structured online questionnaire measuring recognition of complications, referral patterns, and familiarity with audiology resources.	Knowledge, Attitudes, Practices	• 44.5% recognised hearing impairment as a complication (vs >94% for kidney dysfunction, retinopathy, obesity, foot infections).<br>• >60% unfamiliar with referral process and screening frequency.<br>• Most referred <20% of patients for audiology.<br>• Over half are unaware of OTC hearing aids.<br>• Conclusion: Knowledge gaps persisted; hearing loss was a lower priority.
Sewell et al. (2024)	USA (Oklahoma; academic medical centre)	Health care providers (physicians, pharmacists, audiologists, physician assistants, nurse practitioners, nurses, others) and patients with diabetes. Patients were predominantly middle-aged/older adults with type 2 diabetes; ethnic distribution reflected Oklahoma population.	Cross-sectional survey / Investigator-developed survey for providers and patients assessing awareness, barriers, and practices.	Knowledge, Attitudes, Practices	• 25.6% of providers identified hearing impairment as a diabetes complication (vs >96% for retinopathy, nephropathy, and foot complications).<br>• Main provider barriers: lack of familiarity with guidelines (57.3%), competing priorities (35.4%).<br>• 21% of patients recognised the ear as affected by diabetes; only 8.1% informed by a provider.<br>• 24.2% of patients had hearing screening vs 96% with regular eye exams.<br>• Conclusion: Low awareness; hearing health not integrated into diabetes care.

Ge et al. (2024)	China (Shanghai; 56 community health centres)	General practitioners (n=1022). Stratified cluster sample; 87.1% response rate.	Cross-sectional survey / Custom 39-item validated KAP questionnaire (0–100 scores); reliability: test–retest coefficient 0.863.	Knowledge, Attitudes, Practices	• Mean knowledge score: 69.9/100; >30% unfamiliar with screening tools.<br>• Attitude score: 66.1/100; 11.6% did not consider ARHL a disease.<br>• Practice score: 59.9/100; only 16.9% routinely screened.<br>• Referrals: 46.7% preferred specialist referral.<br>• Conclusion: GPs showed low ARHL knowledge, limited attitudes, and poor practices; knowledge linked to better outcomes.
Jinabhai (2023)	South Africa (nationwide)	Medical practitioners (n=236). Diverse workforce; 47% private sector; 48.7% >20 years' experience.	Descriptive cross-sectional survey / Online SurveyMonkey (18 items, 3 sections); validated (I-CVI >79%).	Knowledge, Practices	• 40.3% aware of DM-related auditory symptoms; 59.7% unaware.<br>• 44.9% unfamiliar with audiologist's role.<br>• 51.5% did not refer patients; 64.9% never counselled.<br>• Significant link between awareness and referrals ($p<0.05$).
Kathy Dowd (2011)	USA (nationwide, via AADE channels)	Diabetes educators (n=453). Majority Certified Diabetes Educators (88%), nurses (43%), dietitians (25%), in hospitals/clinics.	Grey literature report; Cross-sectional online survey / Investigator-developed 39-item questionnaire (SurveyMonkey).	Knowledge, Attitudes, Practices	• >51% not aware or only somewhat aware of diabetes–hearing link.<br>• 95% rarely/never referred patients for hearing screening.<br>• 98% did not use screening tools; 72% had little/no awareness of tools.<br>• 40% expressed willingness to use screening tools if trained.<br>• Conclusion: Limited awareness and practices in 2011, but interest in training was evident.
